# Emerging Place of JAK Inhibitors in the Treatment of Inborn Errors of Immunity

**DOI:** 10.3389/fimmu.2021.717388

**Published:** 2021-09-17

**Authors:** Jérôme Hadjadj, Marie-Louise Frémond, Bénédicte Neven

**Affiliations:** ^1^Department of Internal Medicine, National Referral Center for Rare Systemic Autoimmune Diseases, Hôpital Cochin, APHP-Centre Université de Paris (CUP), Paris, France; ^2^Université de Paris, Institut Imagine, INSERMU1163, Laboratory of Immunogenetics of Pediatric Autoimmuninity, Paris, France; ^3^Pediatric Hematology-Immunology and Rheumatology Department, APHP-Centre Université de Paris (CUP), Necker Hospital, Paris, France; ^4^Université de Paris, Institut Imagine, Laboratory of Neurogenetics and Neuroinflammation, Paris, France

**Keywords:** Jakinib, inborn errors of immunity (IEIs), interferonopathies, STAT 1 and STAT3 gain of function, autoimmunity, novel therapies

## Abstract

Among inborn errors of immunity (IEIs), some conditions are characterized by inflammation and autoimmunity at the front line and are particularly challenging to treat. Monogenic diseases associated with gain-of-function mutations in genes critical for cytokine signaling through the JAK-STAT pathway belong to this group. These conditions represent good candidates for treatment with JAK inhibitors. Type I interferonopathies, a group of recently identified monogenic auto-inflammatory diseases characterized by excessive secretion of type I IFN, are also good candidates with growing experiences reported in the literature. However, many questions remain regarding the choice of the drug, the dose (in particular in children), the efficacy on the various manifestations, the monitoring of the treatment, and the management of potent side effects in particular in patients with infectious susceptibility. This review will summarize the current experiences reported and will highlight the unmet needs.

## Introduction

Use of next-generation sequencing (NGS) has permitted the identification of a growing number of inborn errors of immunity (IEIs), with more than 450 conditions following the latest classification from the International Union of Immunological Societies Committee on Inborn Errors of Immunity (1, 2). The clinical features are broad, ranging from increased susceptibility to infections to immune dysregulation, including multiple autoimmune manifestations, allergy, and lymphoproliferation. Increased susceptibility to malignancy can also occur. Several monogenic autoimmune and/or auto-inflammatory diseases have been identified, and the analysis of these genes has provided a wealth of information on the mechanisms of tolerance that control reactivity to self in humans. These last years, monogenic diseases affecting cytokine signaling and in particular the Janus kinase (JAK)/Signal transducer and activator of transcription (STAT) pathway were described (3–7) and responsible for overwhelming human immune dysregulation. These conditions, as well as the hematological diseases related to somatic gain-of-function mutations of JAK2 and STAT3 respectively in myeloproliferative diseases (8) (MPNs) and large granular leukemia (9) (LGL), illustrate the key role of this signaling pathway in the regulation of the immune response (**Table 1**). Furthermore, identification of these mutations may influence treatment options to offer targeted treatment. The goal of this review is to report the available data on drug inhibiting the JAK-STAT pathway, i.e., JAK inhibitor (JAKinib), in the context of IEIs.

**Table 1:** Main IEIs and hematologic conditions in humans related to germline and somatic mutations in members of the JAK-STAT pathway.

mol.	Main signaling pathways	Mode of inh.	LOF	Mode of inh.	GOF
**JAK1**	Gamma chain family, TSLP, GP130 family, IFN I, II, III, IL-10 family	AR	MSMD, mild viral infections	Somatic, AD	Hypereosinophilia, autoimmunity
**JAK2**	Beta chain family, TSLP, GP130 Family, Leptin, GH, prolactin, EPO, TPO, IFN II, IL12 family			Somatic, AD	MPNs
**JAK3**	Gamma chain family	AR	severe combined immunodeficiency	Somatic	T cell malignancies
**Tyk2**	IFN I, III, GP 130 family, IL-10 family, IL-12 family, IL-27	AR	MSMD, intracellular bacteria, viral susceptibility		
**STAT1**	IFN type I, II, III, and IL-27	AR complete	viral infections (mostly herpes virus) mycobacterial infections	AD	CMC, autoimmunity, inflammation
		AR partial	milder disease (same spectrum than AR complete deficiency)		
		AD	MSMD		
**STAT2**	IFN I and III	AR	viral susceptibility including to life vaccines	AR	Early-onset auto-inflammation
**STAT3**	Signaling from a large array of receptors (mainly common beta chain and GP-130 families)	AD^a^	hyper IgE syndrome with CMC and extra-hematopoietic manifestations	Somatic	LGL
				AD	Lympho-proliferation, autoimmunity
**STAT5B**	IL-2, GH	AR	Short stature, autoimmunity, allergy, infectious susceptibility	Somatic	Hypereosinophilia, urticaria, atopic dermatitis, diarrhea

a Dominant negative LOF mutations.

AD, autosomic dominant; AR, autosomic recessive; CMC, chronic mucocutaneous candidiasis; EPO, erythropoietin; IFN, interferon; GH, growth hormone; GOF, gain of function; LGL, large granular leukemia; LOF, loss of function; Mol, molecules; Mode of inh, mode of inheritance; MPNs, myeloproliferative neoplasia; MSMD, Mendelian susceptibility to mycobacterial diseases; TPO, thrombopoietin; TSLP, thymic stromal lymphopoietin.

Beta chain cytokine family: IL-3, IL-5, and GM-CSF; gamma chain cytokine family: IL-2, IL-4, IL-7, IL-9, IL-15, and IL-21; GP130 cytokine family: IL-6, IL-11, IL-31, Oncostatin, ciliary neurotrophic factor, cardiotropin-1, leukemia-inhibitor factor, neurotrophin-1; IFN: interferon type I (alpha and beta), type II (gamma), type III (lambda); IL-10 cytokine family: IL-10, IL-19, IL-20, IL-22, IL-24, IL-26; IL-12 cytokines family: IL-2, IL-13, IL-23.

### The Canonical JAK-STAT Pathway

The JAK-STAT signaling pathway is a direct, evolutionary conserved pathway allowing quick signaling from membrane to nucleus (10, 11). Numerous cytokine, interleukin, hormone, and growth factor (57 in total) signaling pathways rely on JAKs (11). These ligands bind to their cognate type I and II receptors, characterized by the lack of their own enzymatic activity that requires intracytoplasmic physically associated JAK tyrosine kinases. Each receptor uses a selective homo- or heterodimer composed of four JAK molecules (JAK1, JAK2, JAK3, TYK2) (10). Every JAK comprises four structural domains (the carboxy-terminal kinase domain, an adjacent pseudokinase domain, an Src homology 2 (SH2)-like domain, and the amino-terminal FERM domain which interacts with the cytosolic tail of the receptor) (10). Following engagement of the receptor, homo- or heterodimers of JAKs phosphorylate each other’s tyrosine residues as well as the intracellular tail of the receptor, creating a docking site that recruits downstream STAT DNA-binding proteins (10). The phosphorylation of STAT mediates dimerization, translocation, and accumulation into the nucleus and DNA binding to regulate gene expression. There are seven mammalian STATs: STAT1, STAT2, STAT3, STAT4, STAT5A, STAT5B, and STAT6.

### Approved JAK Inhibitors

The implication of cytokines in many autoimmune diseases and the role of JAK2 GOF (gain of function) mutations in MPNs supported the rationale for development of molecules that block the kinase activity of JAKs, preventing phosphorylation of STAT (11).

Because JAKs are essential for signaling downstream from a wide range of substrates, the action of JAK inhibitors (JAKinibs) is large (12). The list of marketed drugs is shown in **Table 2**. Three JAKinibs of the first generation with poor specificity and a large range of cytokines signaling inhibition are approved in humans to treat autoimmune, inflammatory, and hematological conditions (13) such as rheumatoid arthritis (RA), psoriasis arthritis, ulcerative colitis (UC), MPNs, and acute graft versus host disease (GVHD) (11). Other sporadic autoimmune and hematological malignancies are good candidates for JAKinibs, and trials have been performed or are ongoing (in alopecia areata, atopic dermatitis, psoriasis, vitiligo, systemic sclerosis, spondyloarthritis, dermatomyositis, lupus, Crohn’s disease, primary biliary cholangitis, autoimmune hepatic disease, and type I diabetes).

**Table 2:** Approved marketed JAKinibs.

Name	Specificity	Approved indications	Elimination
Tofacitinib	JAK1/JAK3/(JAK2)	RA, PsA, UC, pA JIA	metab. by Cyto.
Baricitinib	JAK1, JAK2	RA	urine excretion
Ruxolitinib	JAK1, JAK2	MPN, acute GVHD	metab. by Cyto.
Peficitinib	pan-JAK	RA (Japan)	metab. indt of cyto
Fedratinib	JAK2, Flt3	MPN	metab. by Cyto.
Upadacitinib	JAK1	RA, PsA	metab. by Cyto.
Filgotinib	JAK1	RA (Europe, Japan)	urine excretion

RA, rheumatoid arthritis; PsA, psoriasis arthritis; UC, ulcerative colitis; pA JIA, polyarticular juvenile idiopathic arthritis; MPN, myeloproliferative neoplasm; GVHD, graft versus host disease; metab. by cyto, metabolized by cytochrome P450 complex; metab. indt of cyto, metabolism independent of cytochrome P450 complex.

Second-generation selective JAKinibs (11), with a more specific anti-JAK-1 (e.g., filgotinib and upadacitinib) or JAK2 (fedratinib) activity, narrow spectrum of action, and possibly improved safety, are now emerging. Topical and inhaled JAKinibs are also under investigation (11).

Most of the marketed JAKinibs are eliminated by metabolism *via* the cytochrome P450 enzymatic complex. This can lead to frequent drug–drug interactions (DDI) that need to be taken into consideration (14). In contrast to the others, baricitinib is mainly cleared by renal elimination through glomerular filtration and active secretion *via* transporters such as OAT3 (15). Thus far, inhibitors of this transporter such as probenecid can increase drug exposition of baricitinib. More broadly, marketed JAKinibs interact with drug transporters in several ways, which may trigger DDI, modify pharmacokinetics (PK), and increase risk of drug toxicities. These DDI are particularly relevant in the field of IEI where co-medications are frequent.

Pharmacokinetic (PK), pharmacodynamic (PD), and dose escalation studies have been performed mainly in adults and have defined the dose and treatment regimen for each JAKinib in approved conditions. FDA has approved (i) tofacitinib in children aged 2 years and older to treat patients with polyarticular juvenile idiopathic arthritis, (ii) baricitinib in children as young as 2 years for emergency use authorization for primary COVID-19 pneumonia in conjunction with remdesivir, and (iii) ruxolitinib down to age 12 for steroid refractory GVHD. PK studies allowed to define dosing regimen in this population for this condition (16). The ruxolitinib PK and preliminary PD phase I study has been performed in children with oncologic or hematologic malignancies with assessment of JAK2 and STAT5 phosphorylation as readout (17). Baricitinib PK and PD studies have been conducted in monogenic interferonopathies in a compassionate use program. Based on these data, a dose and scheme based on body weight and glomerular filtration rate have been proposed to optimize decrease of interferon (IFN) biomarkers (18).

### Side Effects of First-Generation JAKinibs Tofacitinib, Baricitinib, and Ruxolitinib

Data from long-term extension studies and meta-analysis of tofacitinib and baricitinib in RA and ruxolitinib in MPNs provide important insight about the safe profile of these drugs, at least in adults (19–23) with infections being the most frequent followed by cytopenia and hyperlipidemia. Risk of side effects may vary depending on the underlying condition, the concomitant immunosuppression, the population (especially the age), and the dose of JAKinib required to control the disease (24).

#### Infections

The most frequent infectious complication regardless of the drug (tofacitinib, ruxolitinib, and baricitinib) and the underlying disease is reactivation of herpes zoster (23, 25). Other serious or opportunistic infections such as bacterial pneumonia, tuberculosis, BK virus nephropathy (24), and toxoplasmosis are also observed but with a lower incidence (26). One case of progressive multifocal leukoencephalopathy related to John Cunningham (JC) virus infection under ruxolitinib has been reported (27). The unexpected low incidence of serious infections may be due to the spared immune function relying on IL1, IL8, IL17, and TNFα with JAK-independent signaling. This may not be true when concomitant immunosuppressants are used in patients with IEIs.

#### Anemia and Leukopenia

Inhibition of JAK2 may be responsible for anemia and thrombocytopenia by interfering with erythropoietin and thrombopoietin signaling (10). Lymphopenia and a decreased number of NK cells can be observed with tofacitinib depending on the dose, likely due to the inhibition of JAK3-dependent T-cell functions.

#### Lipid and Cardiovascular Diseases

A warning signal for increased risk of thromboembolic complications such as deep vein thrombosis and pulmonary embolism was raised by post-marketing safety studies of tofacitinib, ruxolitinib, and baricitinib, especially in patients carrying other risk factors for such complications (28, 29). These risks appear to be relatively low and might be disease-specific and dose-dependent. Increased low-density lipoprotein cholesterol and triglycerides are observed under JAKinib. Weight gain and increased body mass index (BMI) are reported with ruxolitinib (30) and tofacitinib (31). Such association with baricitinib is less clear. These side effects could be related to reduced postprandial leptine signaling due to JAK2 inhibition resulting in hyperphagia and contributing to weight gain, as demonstrated in mice (30).

#### Cancer

JAKinibs might interfere with adaptive immune function in cancer immunosurveillance and with antineoplasic function of IFN. Thus far, data from a clinical trial in patients with RA or other immune-mediated diseases have not revealed an increased risk of hematological malignancies or solid tumors (32–34). A post-marketing surveillance study for tofacitinib conducted in >4,000 patients (50 years and older) with RA may suggest an increased risk of cancer (35). Additional long-term monitoring and caution is thus required. Long-term data in children are missing.

#### Screening Before Treatment

Independently to disease-specific recommendations, the following screening is advised before JAKinib initiation: complete blood count (CBC), liver function test, serum creatinine, fasting lipid panel, and screening for tuberculosis (36). BK viremia should also be excluded before treatment and monitored since BK nephropathy has been reported in patients treated for interferonopathies (24). Hepatitis B and C and HIV serology may be considered. Vaccination update prior to initiation of treatment is recommended, including varicella vaccine in case of negative serology and if the patient’s immune status allows for live vaccines. The latter will be contraindicated once treatment is initiated.

#### JAKinib Withdrawal Syndrome

Ruxolitinib discontinuation syndrome was mainly reported in patients treated for MPNs (37–39). It is a life-threatening condition characterized by acute relapse of disease symptoms, sometimes mimicking septic shock that occurs 24 h to 3 weeks after drug cessation. To explain this withdrawal syndrome, it was shown that ruxolitinib blocked the dephosphorylation and ubiquitin degradation of JAK1 and JAK2, which accumulated and could lead to a notable activation of downstream signaling when ruxolitinib was removed (39). Similarly, we observed severe relapse of symptoms in patients with monogenic interferonopathies under ruxolitinib when treatment was temporarily stopped (references (40, 41) and unreported observations). Among baricitinib phase 3 trials in patients with RA, a brief interruption of baricitinib was associated with a minor increase of RA symptoms (42) while rebound phenomena were reported in animal models following abrupt withdrawal of JAK1 inhibitor oclacitinib (43) (a first-generation JAKinib prescribed in dogs). The risk of discontinuation syndrome may vary depending on the condition treated and the JAKinib prescribed, but it should not be overlooked. This also indicates the need for a careful tapering of the drug when JAKinib is interrupted.

#### Special Consideration for Children

Growth hormones signal through the JAK-STAT pathway *via* JAK2 (10). The JAK-STAT pathway is also involved in bone homeostasis in various ways since many cytokines with bone-protective and bone-degrading properties signal through JAK-STAT. Of note, Adam et al. showed in various models of mice at steady states and in inflammatory conditions that tofacitinib and baricitinib displayed a bone-sparing effect (44).

### IEI Candidates for JAKinibs, Reported Experiences

**Figure 1:**
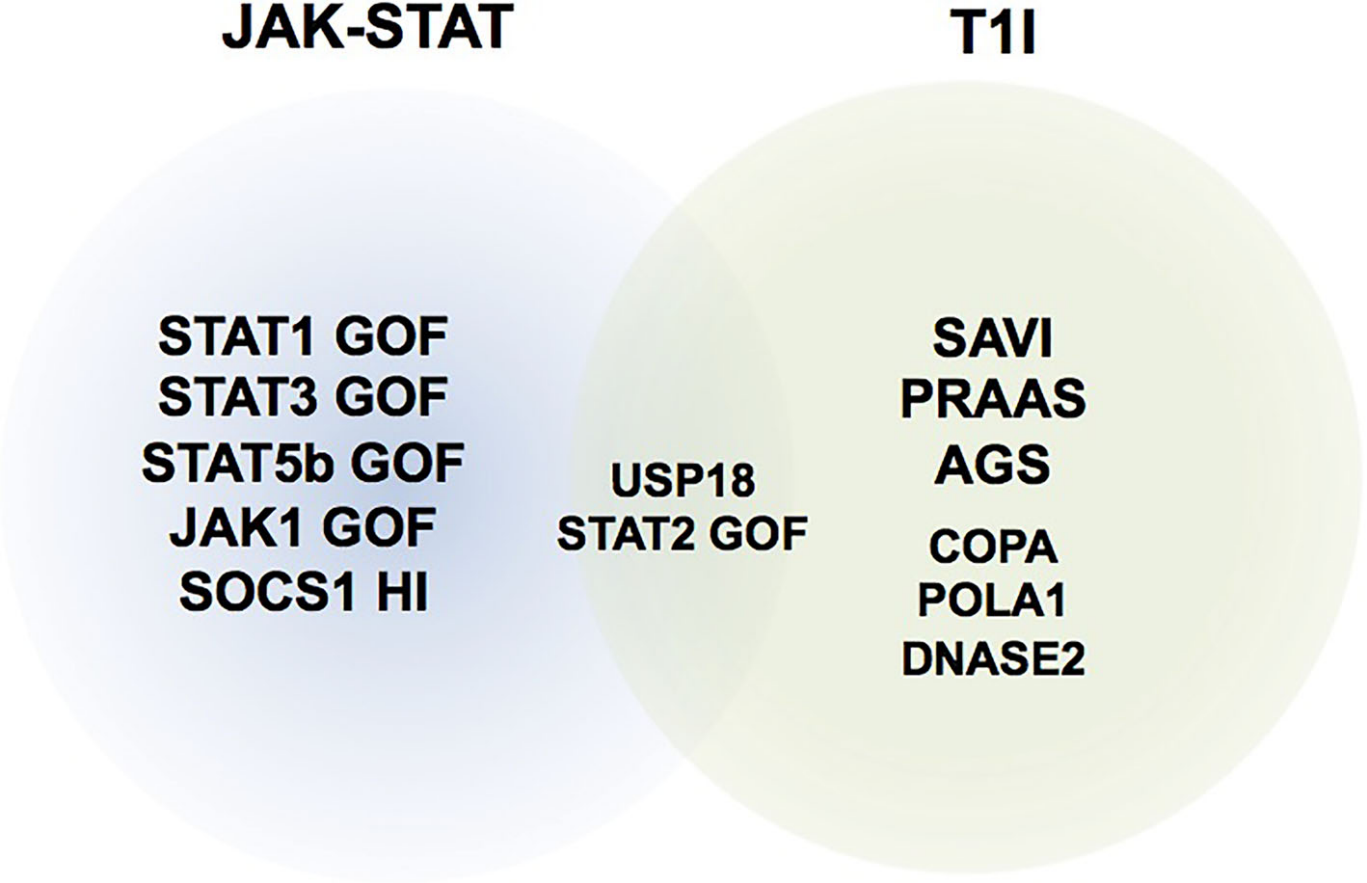
Representative schematic of inborn errors of immunity (IEI) where JAK inhibitors have been used. T1I, type I interferonopathies; JAK-STAT, IEIs related to mutations in components of the JAK-STAT pathway. HI, haploinsufficiency.

These last years, monogenic diseases associated with gain of function of cytokine signaling members and in particular involving the JAK-STAT pathway have been described to cause overwhelming immune dysregulation conditions (**Table 1**). These diseases represent good candidates for treatment with JAKinibs (**Figure 1**). IEIs associated with overproduction of cytokine signaling by the JAK-STAT pathway such as interferonopathies—a group of recently identified monogenic auto-inflammatory diseases characterized by excessive secretion of type I IFN—are also good candidates with growing experiences reported in the literature. All published patients are summarized in the **Supplementary Table 1**. In addition, JAKinibs are promising in primary hemophagocytic lymphohistiocytosis (HLH) (45), a family of diseases characterized by hyper-immune activation and massive release of inflammatory cytokines, mostly but not exclusively IFNγ. Because this topic will be covered in the framework of this series of mini-review, it will not be addressed here.

### STAT1 Gain of Function

Heterozygous *STAT1* mutations that confer hyper-responsiveness to stimulation are associated with a mixed phenotype characterized by infection, autoimmunity, and inflammation. Chronic mucocutaneous candidiasis (CMC) occurs in almost all patients, but a broader infectious susceptibility to invasive fungal infections and bacterial and viral infections is frequent (3, 46, 47). Various autoimmune and inflammatory manifestations are reported in one-third of the patients such as autoimmune endocrinopathies, hepatitis, cytopenia, skin involvement, and pseudo-IPEX enteropathy (48). Vascular aneurysms further enlarge the phenotype. The severity of the disease is variable with a broad spectrum. The increased risk of autoimmunity in these patients might be explained by an enhanced transcription of IFN-induced genes due to signal-induced increased levels of phosphorylated STAT1. CMC can be explained by a decreased proportion of circulating TH17 cells, but the molecular mechanism accounting for this decrease remains to be elucidated (49). Management of these patients included long-lasting antifungal and antibacterial prophylaxis and, when indicated, immunoglobulin replacement. Use of immunosuppression in case of autoimmunity needs to be balanced by the increased infectious susceptibility, and many treatments have failed. Experiences with hematopoietic stem cell transplantation (HSCT) are limited and associated with high morbidity and mortality (50, 51). Use of JAKinib was first reported in 2015 by Higgins et al. in an adult patient suffering from alopecia areata and CMC in whom ruxolitinib treatment allowed complete resolution of both (52). The latter relapsed after cessation of the drug. Since this first promising observation, 11 reports (see **Supplementary Table 1**) shared their experiences with JAKinibs in 18 patients suffering from STAT1 GOF (12 children from 1.1 to 17 years of age and 6 adults), mainly treated by ruxolitinib (n = 17) and baricitinib (n = 1). Forbes et al. reported the largest multicentric retrospective series of 11 patients (53). All patients had experienced CMC or other chronic fungal infections associated with recurrent or chronic viral and/or bacterial infections. Inflammation (mainly inflammatory bowel disease and chronic ulcers) and various autoimmune diseases (autoimmune cytopenia, aplastic anemia, hepatitis, type I diabetes, thyroiditis, etc.) were noticed in 9 and 10 patients respectively. Dysimmune manifestations required several lines of immunosuppressive drugs with limited benefits in most patients. Doses of ruxolitinib were variable, from 0.3 to 1 mg/kg (in two divided doses) in children and from 5 to 40 mg (in one or two divided doses) with a median follow-up of 5 months (range 0.5 to 14). Significant improvement of all main features of the disease was noticed. CMC resolved or improved while chronic severe dermatophytosis and disseminated coccidioidomycosis reported in one patient each got worse (54). Inflammatory and autoimmune manifestations dramatically improved including one teenage patient with reversible type 1 diabetes under ruxolitinib allowing long-lasting remission (55). The benefit of ruxolitinib on T_H_17 cell differentiation or IL-17 production remains controversial (49). Despite these very encouraging clinical results, many questions and uncertainties remain. The follow-up of the reported patients is short, and the optimal dose especially in children and the long-term effects in this particular IEI are unknown. The management of drug interactions of ruxolitinib with drugs metabolized, inhibiting or enhancing similar cytochrome metabolic pathways, in particular azoles and azithromycin, needs special consideration.

### STAT3 Gain of Function

STAT3 hyperactivity due to germline heterozygous GOF mutations causes an early-onset immune dysregulation syndrome characterized by lymphoproliferation and a broad spectrum of autoimmune manifestations (4). Clinical phenotype is variable in terms of organ involved, age at onset, and severity. No clear genotype–phenotype correlation is described. In a systematic review describing clinical aspects of 42 patients from 18 publications, hematologic diseases was the most frequent finding (83% of cases), especially autoimmune cytopenia (56). Immunodeficiency with infection susceptibility and hypogammaglobulinemia were observed in 67% of cases. Other features included type I diabetes, enteropathy, interstitial lung disease (ILD) that can be life-threatening (56), arthritis, and growth failure.

Molecular consequences of STAT3 GOF mutations are variable (57). Autoimmunity might be caused by increased STAT3 activity, especially in response to IL-6 and IL-21, resulting in enhancement of T_H_17 cell fate determination (4, 58). It has also been suggested that reduction in regulatory T cell number and function caused by impaired cytokine-induced phosphorylation of STAT5 could lead to autoimmune manifestation (4).

Before the identification of STAT3 GOF mutations, patients were treated with years of various nonspecific immunosuppressive agents that were overall ineffective. Use of an anti-IL-6 receptor antibody (i.e., tocilizumab) was first reported (4), showing promising results, but the effect seems to plateau. Some patients received HSCT with high mortality (56). Experiences of JAKinibs were published in 13 patients in 6 reports suffering from STAT3 GOF (children from 1 month to 15 years of age) (53, 59–62), in combination with tocilizumab in 6 cases. Indications were mainly lymphoproliferation, autoimmune cytopenia, enteropathy, ILD, and arthritis not controlled with other therapies. Ten patients received ruxolitinib and three tofacitinib with various dosages. Ten patients had significant clinical improvement with a spectacular effect in some cases, i.e., cessation of oxygen therapy (including mechanical ventilation) and radiographic resolution of ILD, or independence of parenteral nutrition for enteropathy (53, 62). Three patients had no or minimal response. In the first case, respiratory failure progressed despite ruxolitinib leading to death. In the second, ruxolitinib was introduced during severe sepsis with multiorgan failure (53). In the last case, indication was pure red cell aplasia and ruxolitinib led to minor improvement (61). Tolerance was good in most cases. Adverse effects included thrombocytopenia, transiently increased transaminase and/or bilirubin levels, and influenza infection. In all cases, the follow-up of the reported patients is short (**Supplementary Table 1**).

### Interferonopathies

Type I interferonopathies are a group of recently identified monogenic auto-inflammatory diseases characterized by constitutive signaling of type I IFN resulting from their excessive or dysregulated secretion. There are more than 25 monogenic diseases that are associated with increased production of type I IFNs (63) which in turn drive the expression of IFN-stimulated genes (ISG) (also called IFN signature) through the engagement of a common receptor that subsequently activates JAK1 and Tyk2. The concept of type I interferonopathies was raised in 2011 and supports the hypothesis that some if not all symptoms of these syndromes would be related to excessive or dysregulated type I IFN production warranting therapeutic intervention with drugs targeting this pathway (64).

#### SAVI

STING-associated vasculopathy with onset in infancy (SAVI) was described in 2014 and is related to GOF mutation in *STING1* (65, 66). Most patients harbor heterozygous mutations, but few patients with homozygous GOF mutation have been recently described (67, 68). STING is a central component in DNA sensing that leads to induction of type I IFNs, which in turn drives the expression of ISG. In a review from December 2020, 70 patients in 49 families are reported (69). The disease is variably characterized by early-onset systemic inflammation with fever, skin vasculopathy, and ILD leading to early-onset pulmonary fibrosis. Arthralgia or arthritis is also frequent, and infections are observed in patients with severe skin lesions and/or lung damage. Chronic elevated inflammatory markers and increased ISG are constant while T cell deficiency with lymphopenia and defect of T cell proliferation are also observed. SAVI is minimally responsive to conventional immunosuppressive therapies and thus is associated with a significant morbidity and increased mortality.

*In vitro*, the three JAKinibs tested on the patient’s lymphocytes (ruxolitinib, tofacitinib, and baricitinib) were able to block the constitutive phosphorylation of STAT1 (65). Based on the hypothesis that inhibiting JAK1 signaling would slow down IFN signaling and thus improve disease-related symptoms, JAKinibs have been proposed to patients suffering from SAVI. To date, 34 patients (14 female) (16 reports) received JAKinibs, i.e., ruxolitinib, baricitinib, and tofacitinib, in 19, 11, and 5 patients, respectively. Patients were less than 2 years in 7 cases and between 2 and 10 years in 13, while 8 were aged 11 to 18 years and 6 were adults. Skin involvement was reported in 22 patients including 9 with severe lesions (ulcers, ischemia of extremities). Lung disease was noticed in all but four patients; arthritis and failure to thrive were reported in eight patients each. All patients with appropriate clinical information provided presented systemic inflammation. Treatment failed on eight occasions, in four patients affected by severe lung involvement and respiratory failure at treatment initiation who died (n = 3) or required lung transplantation (n = 1), in two patients treated with a low dose of ruxolitinib (absence of response), and two patients worsened/deteriorated under tofacitinib (70, 71). Among the remaining patients, skin involvement improved in all cases with complete or partial remission, depending on the initial degree of severity. Lung involvement was also improved, and arthritis mostly resolved. General status and quality-of-life improvements were noticed by all authors. When reported, ISG were not normalized under treatment (24, 40, 41). Doses of the JAKinibs were extremely variable, from 0.2 to 1.5 mg/kg/day for ruxolitinib (10 to 35 mg/m^2^/day) in two divided doses, and from 2.5 to 5 mg bid for tofacitinib. Doses and scheme of administration of baricitinib were supported by PK studies associated with *in vitro* assessment of IFN biomarkers and more homogeneous (18). The main reported side effects were infectious (shingle, rhinovirus, and other viral respiratory infection, rotavirus enteritidis). Aspergilloma in lung cavities was also noticed (reference (41) and unpublished observations). Papillary edema and ruxolitinib discontinuation syndrome were also reported (40, 41).

#### AGS and FCL

Aicardi-Goutières syndrome (AGS) is the paradigm of the type I interferonopathies and is associated with high morbidity and mortality related to the prominent central nervous system involvement. AGS can be caused by any of the nine AGS-related genes (63, 72), with all the proteins encoded involved in either the processing or the sensing of nucleic acids. Given the marked efficacy of JAK1/2 inhibition in chilblain lupus due to TREX1 deficiency (73, 74) or STING GOF, the potential benefit of this treatment on the neurological component of AGS was also considered, despite uncertainty about the bioavailability of the drug in the central nervous system (75–78). Last year, the group of Adeline Vanderver published a large open-label study involving 35 AGS patients treated with baricitinib over a minimal period of 12 months (79). A clear efficacy was observed on skin vasculopathy, and neurological improvement was reported, although evaluation of neurological function is challenging especially in patients with differential onset and disease progression. Related to this, Neven et al. (78) reported an AGS child who presented first neurological symptoms at 14 months, despite that treatment with ruxolitinib started at age 5 months when the child was asymptomatic. This raises the question of (i) the drug penetration in the central nervous system [concentration of ruxolitinib in the cerebrospinal was measured at 10% of that in the blood (78)] and (ii) the role of additional triggers of the disease (e.g., infections and vaccinations). We note here that careful monitoring of pulmonary hypertension is recommended in patients treated with JAKinib considering the high risk of this AGS-related complication (79, 80) that can be exacerbated by JAK inhibition (81).

#### COPA

Heterozygous mutations in the gene encoding the coatomer subunit alpha (COPA) were described in 2015 to underlie an auto-inflammatory disorder associating mainly ILD and/or diffuse alveolar hemorrhage, joint involvement, and lupus-like nephritis (82). The disease is rare (less than 70 patients reported) and characterized by a high frequency of clinical non-penetrance (up to 25%) (69). COPA is part of a complex (COPI) involved in the intracellular trafficking of cargo proteins (83), and mutations in *COPA* were associated with enhanced endoplasmic reticulum (ER) stress and priming of a T_H_17 response (82). More recently, a positive IFN signature was recorded in the blood of several COPA patients (84). Studies from four different teams demonstrated that mutations in *COPA* led to STING-mediated IFN signaling and defined a role for wild-type COPA in STING retrieval from the Golgi back to the ER to prevent chronic immune activation (85–88). Considering these data, a few COPA patients (five in total) have been treated with JAKinib (85, 89–91) and follow-up on treatment has been reported for 3 (89–91). Complete or partial remission has been achieved for arthritis in two patients (89, 91) while a major improvement was observed in a COPA patient with a severe diffuse alveolar hemorrhage (90). However, she has subsequently experienced recurrences of diffuse alveolar hemorrhage associated with progression toward lung fibrosis on chest CT scan (authors’ personal observation).

#### PRAAS

In the large observational study of the use of baricitinib in type I interferonopathies published by Sanchez et al. (24), 10 patients with PRAAS (previously referred to as CANDLE-Chronic Atypical Neutrophilic Dermatosis with Lipodystrophy and Elevated Temperature), harboring monogenic mutations in *PSMB8* (n = 6) and *PSMB4* (n = 1), or digenic mutations in *PSMB4/PSMB9* (n = 2) and *PSMA3/PSMB8* (n = 1), were treated, with a major clinical improvement as compared to SAVI. Indeed, half of the patients reached durable remission with no disease symptoms, normalization of inflammatory markers, and discontinuation of steroid therapy (24). Interestingly, these five patients (who achieved remission) also normalized their IFN score, a unique observation to date in the literature in monogenic IFN-related diseases treated with JAKinib. Of note, one PRAAS patient discontinued the treatment due to acute renal injury related to BK virus infection and subsequently died after a relapse of his disease and a respiratory tract infection. Nevertheless, these very encouraging results in PRAAS were further confirmed by two single patient cases treated with another JAKinib (tofacitinib) (91, 92).

#### Post IFN Signaling

Defective negative regulation of the type IFN response, for example due to LOF (loss of function) mutations in *USP18* and more recently GOF mutations in *STAT2* (93, 94), supports the concept of type I interferonopathies. LOF mutations in USP18 have been described in six patients from three unrelated families (95, 96) to cause pseudo-TORCH syndrome, a severe condition mimicking the phenotype secondary to transplacental transmission of pathogens referred to as TORCH (97). USP18 is recruited by STAT2 to the type IFN receptor subunit IFNAR2 where it competes with JAK1 to enable negative-feedback control of type I IFN signaling (98). Ruxolitinib was trialed in one neonate with inherited USP18 deficiency and was associated with a promising complete recovery with 2 years of follow-up (96).

STAT2 homozygous GOF mutations were reported in three patients from two unrelated families characterized by a severe early-onset inflammation of type I interferonopathies (93, 94). This novel disease largely phenocopies USP18 deficiency in clinical presentation and molecular mechanism by an impaired regulation of late cellular response to type I IFN. Ruxolitinib was used in two cases and led to partial response, but these patients died despite this treatment (93).

The use of JAKinib in these rare inherited diseases with impaired IFN signaling regulation deserves additional reporting.

#### Others

Few other case reports have described the use of JAKinib in the context of monogenic type I interferonopathies [i.e., due to *DNASE2* (91, 99) and *POLA1 *(100) mutations, respectively]. These single observations together with a short follow-up do not allow any conclusion to be reached in these severe disorders, of which the pathophysiology is not yet completely understood.

#### Emerging IEIs

JAK1 GOF mutations leading to activation of multiple STAT proteins were recently reported in four patients from two unrelated families (5, 6). These mutations give rise to a complex immune dysregulatory syndrome characterized by severe atopic dermatitis, profound eosinophilia with eosinophilic organ infiltration, failure to thrive, and autoimmune manifestations such as membranous nephropathy and thyroiditis. The description of this complex phenotype may reflect the scope of the various cytokine signaling pathways involved. JAKinib (ruxolitinib in two cases and tofacitinib in one case) resulted in remarkable improvement in clinical disease and biological abnormalities.

Germline LOF heterozygous SOCS1 mutations leading to haploinsufficiency were also recently described to be associated with a dominantly inherited predisposition to early-onset autoimmune disease including especially autoimmune cytopenias and systemic lupus, related to cytokine hypersensitivity (i.e., IFNγ, IL-2, and IL-4) in immune cells (7, 101). Given that these mutations were associated with uncontrolled JAK-STAT activation after cytokine stimulation, JAK1/2 inhibition was trialed and showed efficacy *in vitro* and *ex-vivo*. Until now, only one patient with SOCS1 deficiency and systemic lupus was treated with JAKinib (baricitinib), showing clinical remission, decrease in anti-DNA autoantibodies, and good tolerance (7). Therefore, JAK inhibitors may represent targeted therapies of value for SOCS1-insufficient patients.

Somatic STAT5B GOF mutations, while frequently described in T lymphocyte-derived neoplasms (102, 103), have been reported in three patients with IEI associating early-onset atopic disease, hyper-eosinophilia, urticaria, dermatitis, and diarrhea (104, 105). Use of ruxolitinib in two patients resulted in remarkable improvement of clinical symptoms and hypereosinophila (105).

## Conclusions and Perspectives

The marketed JAKinib as well as the new more specific inhibitors in development find numerous indications in the field of IEIs, whether related to an intrinsic defect of the JAK-STAT pathway [mutations of one of its components or of a molecule involved in its regulation (i.e., SOCS1)] or extrinsic due to hypersecretion of one or more cytokines signaling through the JAK-STAT pathway. The clinical and biological data of JAKinibs in these conditions summarized above are very promising and open interesting perspectives, but remain preliminary, sporadic, and too heterogeneous to give firm and definitive therapeutic recommendations. Although this analysis does not raise red flags in terms of safety, particularly in terms of infection, caution remains in the context of IEIs. These data highlight the need for prospective, if possible multicenter, evaluation, which would provide answers to the most burning questions: i) what are the risk of infections in the short and long term? ii) what is the long-term safety of JAKinibs in children, particularly with regard to growth and bone metabolism? iii) are these molecules equivalent in terms of safety and efficacy? iv) how does this targeted treatment fit into the management of these conditions? Is it a long-term treatment or a bridge to transplant? v) what dosage and what administration scheme should be proposed according to the pathology and the age of the patient? Should the benefit of the treatment be monitored clinically and/or biologically, and what are the best readouts? The issue of blood–brain barrier crossing will also be important to consider in the context of AGS and HLH. Finally, the benefit of next-generation JAKinibs will need to be assessed. There are obviously more questions than answers, and everything must be done in the coming years to collectively provide answers to these matters.

## Author Contributions

All authors contributed to the article and approved the submitted version.

## Funding

M-LF received a grant from the Institut National de la Santé et de la Recherche Médicale (reference: 000427993) and acknowledges La Fondation Square.

## Conflict of Interest

The authors declare that the research was conducted in the absence of any commercial or financial relationships that could be construed as a potential conflict of interest.

The reviewer AW declared a past co-authorship with the author BN to the handling editor.

## Publisher’s Note

All claims expressed in this article are solely those of the authors and do not necessarily represent those of their affiliated organizations, or those of the publisher, the editors and the reviewers. Any product that may be evaluated in this article, or claim that may be made by its manufacturer, is not guaranteed or endorsed by the publisher.
