# Supplementary material for: Emerging Place of JAK Inhibitors in the Treatment of Inborn Errors of Immunity
Source: Front Immunol. 2021 Sep 17;12:717388. doi: 10.3389/fimmu.2021.717388 (PMC8484879; doi:10.3389/fimmu.2021.717388)
Supplement: Supplementary file 1 [file Table_1.docx]

| **First author** | **Journal** | **Year of publication** | **Disease / Genotype** | **Number of patients** | **Age (range, year)** | **JAK inhibitor** | **Follow-up (range)** |
| --- | --- | --- | --- | --- | --- | --- | --- |
| Higgins | J Allergy Clin Immunol | 2015 | STAT1 GOF | 1 | 28 | Ruxolitinib | 6 months |
| Mossner | Clin Infectious disease | 2016 | STAT1 GOF | 1 | 36 | Ruxolitinib | 5 months |
| Weinacht | J Allergy Clin Immunol | 2017 | STAT1 GOF | 1 | 10 | Ruxolitinib | 12 months |
| Zimmerman | OFID | 2018 | STAT1 GOF | 2 | 27-42 | Ruxolitinib | 1-5 months |
| Meesilpavikkai | J Allergy Clin Immunol | 2018 | STAT1 GOF | 1 | 24 | Baricitinib | 8 months |
| Bloomfield | J Clin Immunol | 2018 | STAT1 GOF | 1 | 12 | Ruxolitinib | 4 months |
| Vargas-Hermandes | J Allergy Clin Immunol | 2018 | STAT1 GOF | 2 (+ 1^£^) | 11-31 |  | 4-29 months |
| Forbes | J Allergy Clin Immunol | 2018 | STAT1 GOF | 7 (+ 3^£^) | 1-31 | Ruxolitinib | 4-29 months |
| Al Shehri | J Clin Immunol | 2019 | STAT1 GOF | 1 | 10 | Ruxolitinib | NA |
| Moriya | J Clin Immunol | 2020 | STAT1 GOF | 1 | 3 | Ruxolitinib | NA |
| Chaimowitz | NEJM | 2020 | STAT1 GOF | 1 | 17 | Ruxolitinib | 27 months |
| Forbes | J Allergy Clin Immunol | 2018 | STAT3 GOF | 6 | 3-15 | Ruxolitinib (n=5) and Tofacitinib (n=1) | 1-18 months |
| Mauracher | J Allergy Clin Immunol | 2019 | STAT3 GOF | 1 | 10 | Ruxolitinib | NA |
| Parlato | Gastroenterology | 2019 | STAT3 GOF | 1 | 0.4 | Ruxolitinib | 12 months |
| Silva-Carmina | AJRCCM | 2020 | STAT3 GOF | 3 | 0.1-1 | Tofacitinib (n=2) and Ruxolitinib (n=1) | 18-29.5 months |
| Wegehaupt | J Clin Immunol | 2020 | STAT3 GOF | 1 | 1.5 | Ruxolitinib | 27 months |
| Sarfati | J Clin Immunol | 2021 | STAT3 GOF | 1 | 0.2 | Ruxolitinib | 6 months |
| Frémond | J Allergy Clin Immunol | 2016 | SAVI / *STING1* | 3 | 5-12 | Ruxolitinib | 6-18 months |
| König | Ann Rheum Dis | 2016 | SAVI / *STING1* | 2 | 58-60 | Tofacitinib | 17 days |
| Manoussakis | Rheumatology | 2017 | SAVI / *STING1* | 1 | 18 | Ruxolitinib | 1 month |
| Saldanha | Front Immunol | 2018 | SAVI / *STING1* | 1 | 2.75 | Ruxolitinib | Not mentioned |
| Sanchez | J Clin Invest | 2018 | SAVI / *STING1* | 4 | 7-24 | Baricitinib | 1.5-4.9 years^#^ |
| Volpi | J Clin Immunol | 2019 | SAVI / *STING1* | 3 | 2-9 | Ruxolitinib | 6-32 months |
| Balci | Cli Immunol | 2019 | SAVI / *STING1* | 1 | 0.8-1.25 | Ruxolitinib switched to baricitinib | 3 weeks/2 months |
| Tang | J Clin Immunol | 2019 | SAVI / *STING1* | 2 | 1.1-5.3 | Tofacitinib | 7-10 months |
| Keskitalo | Front Immunol | 2019 | SAVI / *STING1* | 1 | 37 | Baricitinib | 3 months |
| Clarke | Ped Rhum | 2020 | SAVI / *STING1* | 2 | 0.7-22 | Baricitinib | Not mentioned |
| Lin | J Allergy Clin Immunol | 2020 | SAVI / *STING1* | 4 | 0.4-7 | Tofacitinib (n=1), Ruxolitinib (n=1), Baricitinib (n=2) | Not mentioned |
| Ma | J Clin Immunol | 2020 | SAVI / *STING1* | 1 | 3 | Baricitinib | Not mentioned |
| Frémond* | J Allergy Clin Immunol Prat | 2020 | SAVI / *STING1* | 5* | 0.5-14 | Ruxolitinib | 2.5-41 months |
| Alghamdi | Front Immunol, | 2021 | SAVI / *STING1* | 2 | 9-15 | Ruxolitinib | 6 months*** |
| Wang | Ann Transl Med | 2021 | SAVI / *STING1* | 2 | 13-37 | Ruxolitinib | 4 months*** |
| Kothur | Neurology | 2018 | AGS / *IFIH1* | 1 | 2 | Ruxolitinib | 19 months |
| McLellan | J Clin Immunol | 2018 | AGS / *IFIH1* | 1 | 7.5 | Ruxolitinib | 2 years |
| Zheng | Front Immunol | 2020 | AGS / *IFIH1* | 1 | 13 | Tofacitinib | 3 months |
| Vanderver | NEJM, 2020 | 2020 | AGS / *AGS1-7* | 35 | 0.2-21.8 | Baricitinib | 11.8-43.8 months |
| Neven | NEJM, 2020 | 2020 | AGS / *RNASEH2B* | 1 | 0.4 | Ruxolitinib | 13 months |
| Zhang | Pediatr Rheum | 2021 | FCL / *TREX1* | 1 | 2 | Tofacitinib | 24 months |
| Briand | Ann Rheum Dis | 2017 | FCL / *TREX1* | 1 | 5 | Ruxolitinib | 12 months |
| Sanchez | J Clin Invest | 2018 | PRAAS | 10 | Unknown | Baricitinib | 1.5-4.9 years^#^ |
| Pin | Int J Mol Sci | 2020 | PRAAS | 1 | 17 | Tofacitinib | Not mentioned |
| Patel | Pediatr Dermatology | 2021 | PRAAS | 1 | 12 | Tofacitinib | 12 months |
| Krutzke | Eur J Rheumatol | 2019 | COPA / *COPA* | 1 | 15 | Baricitinib | 12 months |
| Frémond | Thorax | 2020 | COPA / *COPA* | 1 | 11 | Ruxolitinib | 12 months |
| Pin | Int J Mol Sci | 2020 | COPA / *COPA* | 1 | 4 | Baricitinib | 15 months |
| Pin | Int J Mol Sci | 2020 | DNASE II / *DNASE2* | 1 | 15 | Ruxolitinib | Not mentioned |
| Légeret | J Clin Immunol | 2020 | XLPDR / *POLA1* | 1 | 11 | Tofacitinib | 8 months |
| Del Bel | J Allergy Clin Immunol | 2017 | JAK1 GOF | 2 | 2-6 | Ruxolitinib | 1 month |
| Gruber | Immunity | 2020 | JAK1 GOF | 1 | 18 | Tofacitinib | 24 months |
| Hadjadj | Nature Com | 2020 | SOCS1 LOF | 1 | 24 | Baricitinib | 4 months |
| Duncan | Science Immunol | 2019 | STAT2 GOF | 2 | 0.2-1.5 | Ruxolitinib | 1-3 months |
| Eisenberg | J Allergy Clin Immunol Prat | 2021 | STAT5b GOF | 2 | 2-4 | Ruxolitinib | 12 and 24 months |

Abbreviations: AGS: Aicardi-Goutières syndrome; FCL: familial chilblain lupus; PRAAS: proteasome-associated autoinflammatory syndrome; SAVI: STING-associated vasculopathy with onset in infancy; XLPDR: X-linked reticulate pigmentary disorder.

^*^The three patients under ruxolitinib already reported in the paper from Frémond et al (JACI 2016) are not included here (although the follow-up is extended).

^£^ patient previously reported

^**^Not mentioned for two patients.

^***^Not mentioned for one patient.

^#^The follow-up is given for all patients reported in the manuscript from Sanchez et al (JCI).
